# A novel compound heterozygous mutation in *TTC8* identified in a Japanese patient

**DOI:** 10.1038/s41439-019-0045-y

**Published:** 2019-03-12

**Authors:** Shigeru Sato, Takeshi Morimoto, Kikuko Hotta, Takashi Fujikado, Kohji Nishida

**Affiliations:** 1 Department of Ophthalmology, Osaka University Graduate School of Medicine, Osaka, Japan; 2 Department of Applied Visual Science, Osaka University Graduate School of Medicine, Osaka, Japan; 3 Department of Medical Innovation, Osaka University Hospital, Osaka, Japan

**Keywords:** Disease genetics, Genetic predisposition to disease

## Abstract

Bardet–Biedl syndrome (BBS), characterized by rod-cone dystrophy, postaxial polydactyly, central obesity, hypogonadism, renal abnormalities, and mental retardation, is a rare autosomal recessive disorder. To date, 21 causative genes have been reported. Here we describe a Japanese BBS patient with a novel compound heterozygous mutation in *TTC8*. To the best of our knowledge, this is the first description of a BBS patient with a mutation in the *TTC8* gene in Japan.

Bardet–Biedl syndrome (BBS) is a rare autosomal recessive disorder characterized by rod-cone dystrophy, postaxial polydactyly, central obesity, hypogonadism, renal abnormalities, and mental retardation. BBS is often complicated by strabismus/cataracts/astigmatism, diabetes mellitus, Hirschsprung disease, heart disease, and/or liver fibrosis. To date, 21 causative genes have been reported, comprising ~80% of BBS genetic abnormalities^1,2^. The remaining 20% of genetic abnormalities among BBS patients are not yet known. In the present study, we performed whole-exome sequencing (WES) of a classical BBS patient.

The patient was diagnosed with BBS at 8 years of age, in accordance with criteria reported previously^3^. Primary and secondary signs of BBS in this patient are listed in Table 1. When the patient first visited Osaka University Hospital at 17 years of age, his best-corrected visual acuity (BCVA) was 0.07 in the right eye and 0.2 in the left eye. At 28 years of age, his BCVA was 0.01 in the right eye and 0.04 in the left eye; he exhibited bilateral diffuse retinal degeneration, including macular atrophy, attenuated retinal vessels, and optic nerve head pallor with little pigmentary dispersion. His parents were not consanguineous. His mother showed no sign of BBS or rod-cone dystrophy. His father did not have symptoms of BBS.

**Table 1:** Primary and secondary signs of BBS in this patient

	Age of onset	Clinical information	Intervention
Primary signs			
Rod-cone dystrophy	8 Years old	Visual acuities: 0.01 (right), 0.04 (left), (with mild myopia and astigmatism)	No medication
		Fundus finding: binocular diffuse retinal degeneration Visual field: centipede constriction (binocular) Optical coherence tomography: binocular diffuse thinning of outer retinal layer ( + ), macular atrophy ( + ), macular edema ( − ), cystic changes ( − ), elipsoid zone ( − ) Fundus autofluorescence: binocular mottled pattern ( + ), perifoveal ring ( − )	
Polydactyly	At birth	Both feet	Plastic surgery (19 months old)
Obesity	9 Years old	Height: 164 cm Weight: 78.1 kg Body mass index (BMI): 29 kg/m^2^	No medication
Hypogonadism		Testosterone: 300–600 ng/dl	No medication
Renal anomalies	1 Week old	Cystic kidney Creatinine: 1.79 mg/dl BUN: 21 mg/dl eGFR cre: 37.2 mL/min/1.73 m^2^	No medication
Mental retardation	No	-	-
Secondary signs			
Hirschsprung disease	3 Months old	-	Surgery (28 months old)
Abnormal glucose tolerance	9 Years old	HbA1c: 5.6%, 75 g oral glucose tolerance test: 82 mg/dL at 0 h, 185 mg/dL at 2 h	No medication
Exotropia	NA	-	Bilateral lateral rectus muscle recession (14 years old)
Hypertension	27 Years old	Blood pressure = 145/83 mm Hg	Oral medicine (Azilsartan 20 mg and Amlodipine besilate 3.47 mg per day)
Cataract	NA	Binocular anterior sub-capsular cataract	-
Heart diseases	No	-	-
Liver fibrosis	No	-	-

All experimental procedures were approved by the Ethics Committee at Osaka University (No. 719–2, Osaka, Japan) and conducted in accordance with the Declaration of Helsinki. Written informed consent was obtained from the patient (at the time of the report, a 28-year-old male) and his 61-year-old mother. Both individuals underwent ophthalmologic examinations: BCVA in decimal units, slit-lamp biomicroscopy, fundoscopy, visual field testing with Goldmann perimetry, optical coherence tomography (SSOCT; DRI OCT1, Topcon Corp., Tokyo, Japan), and fundus autofluorescence (Optos, Optos KK, Tokyo, Japan). Genomic DNA was extracted from blood samples using NucleoSpin Blood XL (Macherey-nagel, Düren, Germany). DNA libraries were constructed using SureSelectXT Human All Exon Kit V6 and SureSelectXT Reagent Kit (Agilent, Santa Clara, CA, USA) and then subjected to 100 bp paired-end sequencing on an Illumina HiSeq2500 Platform (Illumina, San Diego, CA, USA). Sequence reads were aligned to the reference human genome (UCSC hg19) in BWA (http://www.bio-bwa.sourceforge.net/) to align short reads after adaptor sequences were removed by Cutadapt (https://cutadapt.readthedocs.io/en/stable/). SAM tools (Version 0.1.17; http://www.samtools.sourceforge.net/) were used for sequence data conversion, sorting, and indexing. To exclude duplicate reads, Picard (http://picard.sourceforge.net) was used. Variants were determined using GATK (http://www.broadinstitute.org/gatk/). ANNOVAR (http://www.openbioinformatics.org/annovar/) was used to annotate the resulting genetic variants. Rare variants (minor allele frequency < 0.05) were selected using the Exome Sequencing Project, 1000 Genomes Project, and Human Genetic Variation databases; possible pathogenic variants, such as nonsynonymous, nonsense, and frameshift mutations, were extracted from among the retinal degenerative disease-related genes registered in the Ret.Net.^TM^ database.

Ten candidate pathogenic rare variants in genes related to retinal degenerative diseases were detected in this patient. All were heterozygous variants; however, two novel nonsense (NM_001288781.1 [TTC8_v001]: c.226 C > T, p.Q76X) and frameshift (NM_001288781.1 [TTC8_v001]: c.309_310insTA, p.T103fs) mutations were located in the *TTC8* gene (also known as *BBS8*). Both mutations were validated by direct sequencing of PCR products (Applied Biosystems 3730 DNA Analyzer; Thermo Fisher Scientific K.K., Tokyo, Japan). The primer sets used for PCR were as follows: c.226 C > T, 5′-TGGGTTTTAGGCAGCTTGGA-3′ and 5′-ACCATAAGGCAGAACAGAAACCA-3′; c.308_309insAT, 5′-TAGGCCCTGGAACGTCTTTG-3′ and 5′- ACCATAAGGCAGAACAGAAACCA-3′. This mutation is likely to be pathogenic, because the *TTC8* gene has been reported as a causative gene for BBS8^4^. The nonsense mutation was located in exon 3 of the *TTC8* gene, thus producing a truncated protein without tetratricopeptide repeats 11 and 15, which are involved in pilus formation and twitching mobility. The frameshift mutation in exon 5 (c.309_310insTA) generates a premature stop codon in exon 6, which also produces TTC8 lacking normal tetratricopeptide repeats 11 and 15. The premature stop codon is located before the last exon; notably, a mRNA transcribed from a gene with a truncating mutation often undergoes nonsense-mediated mRNA decay before translation^5^. Thus, transcripts with nonsense and frameshift mutations are likely to be rapidly degraded to reduce the translation of the truncated TTC8 protein. Therefore, this compound heterozygous patient would not have a functional TTC8 protein to support the formation of the BBSome, leading to the development of BBS. His mother exhibited the heterozygous nonsense mutation, but no frameshift mutation. Although the genetic and clinical data were not available from his father, this patient’s BBS was determined to result from a compound heterozygous *TTC8* gene mutation.

BBS patients with mutations in the *TTC8* gene comprise only 2.8% of all BSS patients^6,7^. In Japan, the genetics of four BBS families have been reported: *BBS2*, *BBS5*, and *BBS7* homozygotes, as well as a *BBS10* compound heterozygote^8,9^. To the best of our knowledge, this is the first BBS patient with a mutation in the *TTC8* gene in Japan. Thus far, 16 families with the *TTC8* genetic abnormality have been reported (Table 2)^4,7,10–15^. Most of these families have homozygous mutations; only our patient and a Hispanic family were compound heterozygotes. Although full clinical information was not available for some cases, most of the cases in these 16 families exhibit classical BBS without obvious differences in phenotypes.

**Table 2:** List of variants and phenotype reported in patients of BBS8

Family	Ethnic	Consanguineous	Gene	Nucleotide alteration(s)	Zygosity state	Alteration(s) in coding sequence	Rod-cone dystrophy	Polydactyly	Obesity	Hypogonadism	Renal anomalies	Mental retardation	Secondary signs	Reference
Family 1	Japanese	No	*TTC8*	226 C > T & 308_309insAT	comp. het	Q76X & T103fs	Yes	Yes	Yes	No	Yes	No	Hirschsprung disease, abnormal glucose tolerance, exotropia, hypertension	Present study
Family 2	Pakistan	Yes	*TTC8*	IVS10 + 2_4delTGC	hom	Splice site	Yes	Yes	Yes	Yes	NA	Speech impediment	Developmental delay, brachycephaly	Ansley et al.^4^
Family 2	Pakistan	Yes	*TTC8*	IVS10 + 2_4delTGC	hom	Splice site	Yes	Yes	Yes	Yes	NA	Speech impediment	Developmental delay, brachycephaly, Situs inversus	Ansley, et al.^4^
Family 2	Pakistan	Yes	*TTC8*	IVS10 + 2_4delTGC	hom	Splice site	Yes	Yes	Yes	Yes	NA	Speech impediment	Developmental delay, brachycephaly, hemophilia	Ansley, et al.^4^
Family 3	Saudi Arabian	NA	*TTC8*	187–188delEY	hom	6 bp Inframe delation	Yes	Yes	Yes	Yes	NA	Speech impediment	Developmental delay, brachycephaly	Ansley, et al.^4^
Family 3	Saudi Arabian	NA	*TTC8*	187–188delEY	hom	6 bp Inframe delation	Yes	Yes	Yes	Yes	NA	Speech impediment	Developmental delay, brachycephaly	Ansley, et al.^4^
Family 3	Saudi Arabian	NA	*TTC8*	187–188delEY	hom	6 bp Inframe delation	Yes	Yes	Yes	NA	NA	Speech impediment	Developmental delay, brachycephaly,deafness	Ansley, et al.^4^
Family 4	Saudi Arabian	NA	*TTC8*	187–188delEY	hom	6 bp Inframe delation	Yes	Yes	Yes	NA	NA	Speech impediment	Developmental delay, brachycephaly,hyposadias	Ansley, et al.^4^
Family 4	Saudi Arabian	NA	*TTC8*	187–188delEY	hom	6 bp Inframe delation	Yes	Yes	Yes	NA	NA	Speech impediment	Developmental delay, brachycephaly,asthma	Ansley, et al.^4^
Family 5	North African	Yes	*TTC8*	459 G > A	hom	Splice site	Yes	Yes	NA	NA	NA	Cognitive impairment	Micropenis	Stoetzel, et al.^7^
Family 5	North African	Yes	*TTC8*	459 G > A	hom	Splice site	Yes	Yes	NA	NA	Yes	NA	Hydrometrocolpos	Stoetzel, et al.^7^
Family 5	North African	Yes	*TTC8*	459 G > A	hom	Splice site	Yes	Yes	NA	NA	Yes	NA	NA	Stoetzel, et al.^7^
Family 6	Lebanese	Yes	*TTC8*	IVS6 + 1_G > A	hom	Splice site	NA	NA	NA	NA	NA	NA	NA	Stoetzel, et al.^7^
Family 7	Caucasian	No	*TTC8*	IVS6 + 1–2delGT	het	Splice site	NA	NA	NA	NA	NA	NA	NA	Stoetzel, et al.^7^
Family 8	Tunisian	NA	*TTC8*	459 + 1 G > A	hom	Pro101LeufsX12	NA	NA	NA	NA	NA	NA	NA	Smaoui, et al.^10^
Family 9	Tunisian	NA	*TTC8*	459 + 1 G > A	hom	Pro101LeufsX12	NA	NA	NA	NA	NA	NA	NA	Smaoui, et al.^10^
Family10*	Tunisian	NA	*TTC8*	355_356insGGTGGAAGGCCAGGCA	hom	Thr124ArgfsX43	NA	NA	NA	NA	NA	NA	NA	Smaoui, et al.^10^
Family 11	Turkey	Yes	*TTC8*	122 G > A	hom	W41X	Yes	Yes	Yes	Yes	No	NA	Yes but details unknown	Harville, et al.^11^
Family 12	NA	NA	*TTC8*	IVS2 + 1 G > A	hom	Splice site	Yes	Yes	Yes?	No	No	Yes	Asthma, nasal cephalocele	Janssen, et al.^12^
Family 13	Hispanic	NA	*TTC8*	485delG & 1000delA	comp. het	G162fsX4 & I334fsX1	Yes	Yes	Yes	Yes	Yes	Yes	Fatty liver, gall stones	Janssen, et al.^12^
Family 14	Tunisian	Yes	*TTC8*	329 G > A	hom	Splice site	NA	NA	NA	NA	NA	NA	NA	Redin, et al.^13^
Family 15	Tunisian	Yes	*TTC8*	459 + 1 G > A	hom	Splice site	Yes	Yes	Yes	Yes	Yes	NA	Dental anomalies, hypertension	M’hamdi O, et al.^14^
Family 16	Pakistan	Yes	*TTC8*	1347 G > C	hom	Gln449His	Yes	Yes	Yes	Yes	No	Congnitive impairment	Clinodactyly	Ullah, et al.^15^
Family 16	Pakistan	Yes	*TTC8*	1347 G > C	hom	Gln449His	Yes	Yes	Yes	Yes	NA	Congnitive impairment	Clinodactyly	Ullah, et al.^15^
Family 16	Pakistan	Yes	*TTC8*	1347 G > C	hom	Gln449His	Yes	Yes	Yes	NA	No	Congnitive impairment	NA	Ullah, et al.^15^

In summary, we identified a novel compound heterozygous mutation in a Japanese BBS patient by WES. Our findings suggest that WES may be a useful tool for genetic diagnosis and characterization of BBS.

## Data Availability

The relevant data from this Data Report are hosted at the Human Genome Variation Database at 10.6084/m9.figshare.hgv.2528; 10.6084/m9.figshare.hgv.2531
